# Remdesivir is efficacious in rhesus monkeys exposed to aerosolized Ebola virus

**DOI:** 10.1038/s41598-021-98971-0

**Published:** 2021-09-30

**Authors:** Travis K. Warren, Christopher D. Kane, Jay Wells, Kelly S. Stuthman, Sean A. Van Tongeren, Nicole L. Garza, Ginger Donnelly, Jesse Steffens, Laura Gomba, Jessica M. Weidner, Sarah Norris, Xiankun Zeng, Roy Bannister, Tomas Cihlar, Sina Bavari, Danielle P. Porter, Patrick L. Iversen

**Affiliations:** 1 United States Army Medical Research Institute of Infectious Diseases, Fort Detrick, Frederick, MD 21702 USA; 2 The Geneva Foundation, Tacoma, WA 98402 USA; 3 Gilead Sciences, Foster City, CA 94404 USA; 4 Present Address: Laulima Government Solutions, Integrated Research Facility NIAID/NIH, Fort Detrick, Frederick, MD 21702 USA; 5 Present Address: National Cancer Institute, Frederick, MD 21702 USA; 6 Present Address: SARS-CoV-2 Virology Core, LVD/NIAID/NIH, Bethesda, MD 20892 USA; 7 Present Address: Thomas Scientific, Swedesboro, NJ 08085 USA; 8 Present Address: Helios, Frederick, MD 21702 USA

**Keywords:** Microbiology, Virology, Ebola virus, Infectious diseases

## Abstract

Efficacious therapeutics for Ebola virus disease are in great demand. Ebola virus infections mediated by mucosal exposure, and aerosolization in particular, present a novel challenge due to nontypical massive early infection of respiratory lymphoid tissues. We performed a randomized and blinded study to compare outcomes from vehicle-treated and remdesivir-treated rhesus monkeys in a lethal model of infection resulting from aerosolized Ebola virus exposure. Remdesivir treatment initiated 4 days after exposure was associated with a significant survival benefit, significant reduction in serum viral titer, and improvements in clinical pathology biomarker levels and lung histology compared to vehicle treatment. These observations indicate that remdesivir may have value in countering aerosol-induced Ebola virus disease.

## Introduction

Filoviruses, such as Ebola virus (EBOV) and Marburg virus (MARV), pose an increasing health risk to humans. Since December 2013, atypically extensive EBOV disease (EVD) outbreaks have profoundly impacted public health systems. At least 13,675 EVD fatalities were reported from December 2013 to April 5, 2020^1,2^. Similar to the current coronavirus disease 19 pandemic; local, national, and international organizations were caught unprepared for the EVD outbreaks because EBOV was considered an exotic pathogen of largely negligible consequence for global public health at the time^3–5^. At present, three immune-based treatments/prophylaxes are available: the rVSVΔG-ZEBOV-GP (Ervebo) vaccine was approved in December 2019, the monoclonal antibody (mAb) cocktail REGN-EB3 (Inmazeb) was approved in October 2020, and the mAb Ebanga (Ansuvimab-zykl) was approved in December 2020^6–8^. No nonimmune-mediated therapy has been approved to date.

EBOV is primarily transmitted by direct human-to-human contact or contact with infected tissues, body fluids, or contaminated fomites^9,10^. Evidence from EVD epidemiology suggest that aerosol transmission of EBOV is unlikely and might be limited to very rare cases of humans exposed to aerosol-creating hospital procedures (e.g., intubation or droplets emitted from patients coughing or sneezing)^11–13^. However, opportunistic aerosol transmission of EBOV is conceivable, particularly with patients in the late stage of disease that are undergoing an exponential increase in viral loads and are likely to be highly infectious while experiencing severe diarrhea, vomiting, and bleeding^10,13^. An aerosol transmission route is biologically plausible for a pathogen when (i) aerosols containing the pathogen are generated by or from an infectious person, (ii) the pathogen remains viable in the environment for some period of time, and (iii) the target tissues in which the pathogen initiates infection are accessible to the aerosol^14^. These conditions are relevant to EBOV as the virus is detectable in the pulmonary alveoli, saliva, stool, feces, blood, and other body fluids of patients that can be aerosolized, for instance through forceful emission of body fluids during severe diarrhea, vomiting, bleeding, or coughing and through health care delivery^15,16^. EBOV also can remain replication-competent in aerosols for approximately 100 min^17^. Additionally, EBOV initiates infection in diverse target tissues, including cells present in the respiratory tract, such as macrophages and epithelial cells^18^.

Plausible transmission of EBOV by small-particle aerosols has been documented in experimental settings using nonhuman primates (NHPs)^19^. Rhesus monkeys exposed to aerosol doses of up to 1,590 plaque-forming units (pfu) of EBOV die 7–10 days post-exposure. Viral RNA is detected in the lungs and tracheobronchial lymph nodes by day 3 post-exposure, and serum viral RNA levels of > 10^6^ pfu/mL are detected on days 4–6 post-exposure. The characteristics of EVD in aerosol exposure (AE) studies in NHPs are nearly identical to the key disease manifestations observed in intramuscular (IM) exposure studies with regard to survival, time to death, viremia peak, and infectious virus profile. The generalized progression of acute EVD following exposure to EBOV by aerogenous or hematogenous routes in rhesus macaques (eg: fever, inflammation, coagulopathy, liver and kidney dysfunction, decreased responsiveness, and ultimately mortality) are concordant. However, some modest differences have been documented: (i) EVD clinical signs, such as such as lymphopenia, thrombocytopenia and macular rush, are observed 1 day earlier after IM exposure (day 5) compared to AE (day 6), (ii) a greater increase in activated partial thromboplastin time (APTT) is observed in IM exposure compared to AE, (iii) a greater decline in platelet number is observed following IM exposure versus AE, and (iv) a greater involvement of EVD in the lung is observed following AE compared to IM exposure^20–24^. Whereas following aerosol exposure direct infection of airway epithelium and lung alveoli is observed in rhesus monkeys, there is typically no evidence of direct viral infection of the lung parenchyma following IM inoculation^25,26^.

In patients, route of infection can influence disease course and outcome^27^. The large surface area of the lung combined with the vulnerability of its single layer of epithelial cells in the alveoli makes the lung a particularly sensitive organ following infection via aerosol. Furthermore, once resident alveolar macrophages become activated by a virus, production of interferons, cytokines, and chemokines recruits and regulates the activation of additional immune and inflammatory cell populations in the lung^28^. This sequence of events, subsequent to virus infection in the alveoli, may impair gas exchange and cause damage to the lung epithelium, resulting in pneumonia.

Considering all of the above, the concern for EBOV to be used as a biological weapon via small-particle aerosols has prompted the characterization of filovirus disease in animal models following AE and has raised the question of whether available medical countermeasures are effective in such scenarios.

To our knowledge, therapeutic efficacy studies have not been published using aerosolized EBOV exposure in NHPs. Remdesivir is a 1ʹ-cyano-substituted adenosine nucleotide analog with antiviral activity against a broad range of viruses. Remdesivir treatment is highly efficacious against filovirus infections both in vitro and in animal models^29–34^. Previously, we have demonstrated that intravenous (IV) remdesivir provides a statistically significant clinical benefit in rhesus monkeys and crab-eating macaques parenterally inoculated with EBOV or MARV when treatment was initiated 4 days after virus inoculation^34,35^. Here we describe the first study to evaluate the efficacy of IV remdesivir in rhesus monkeys exposed to aerosolized EBOV.

## Results

### Experimental design

All 12 rhesus macaques were exposed by aerosol delivery to a target dose of 100 pfu EBOV resulting in a calculated titer of exposure ranging from 56 to 118 pfu (average titer was 78 pfu). The randomized and blinded study involved two groups of six. Each group was gender-balanced with three males and three females. In one group, remdesivir was administered once a day IV beginning on day 4 post-inoculation (PI) at a dose of 10 mg/kg and continuing for 11 subsequent days at a dose of 5 mg/kg. In the second group, animals received matched drug vehicle (Table 1) according to the same schedule.

**Table 1:** Study design. *EBOV* Ebola virus,

Group number	Number males/number females	Treatment, dose	Treatment route, frequency	Treatment duration	Challenge
1	6 (3 M/3F)	Vehicle	IV, Once daily	Days 4–15: Vehicle	EBOV head-only exposure to aerosolized virus (100 pfu)
2	6 (3 M/3F)	Remdesivir 10/5 mg/kg	IV, Once daily	Day 4: Remdesivir 10 mg/kg Days 5–15: Remdesivir 5 mg/kg	

*F* female, *IV* intravenous, *M* male, *pfu* plaque-forming units, *PI* post-inoculation (with virus).

### Survival

**Figure 1:**
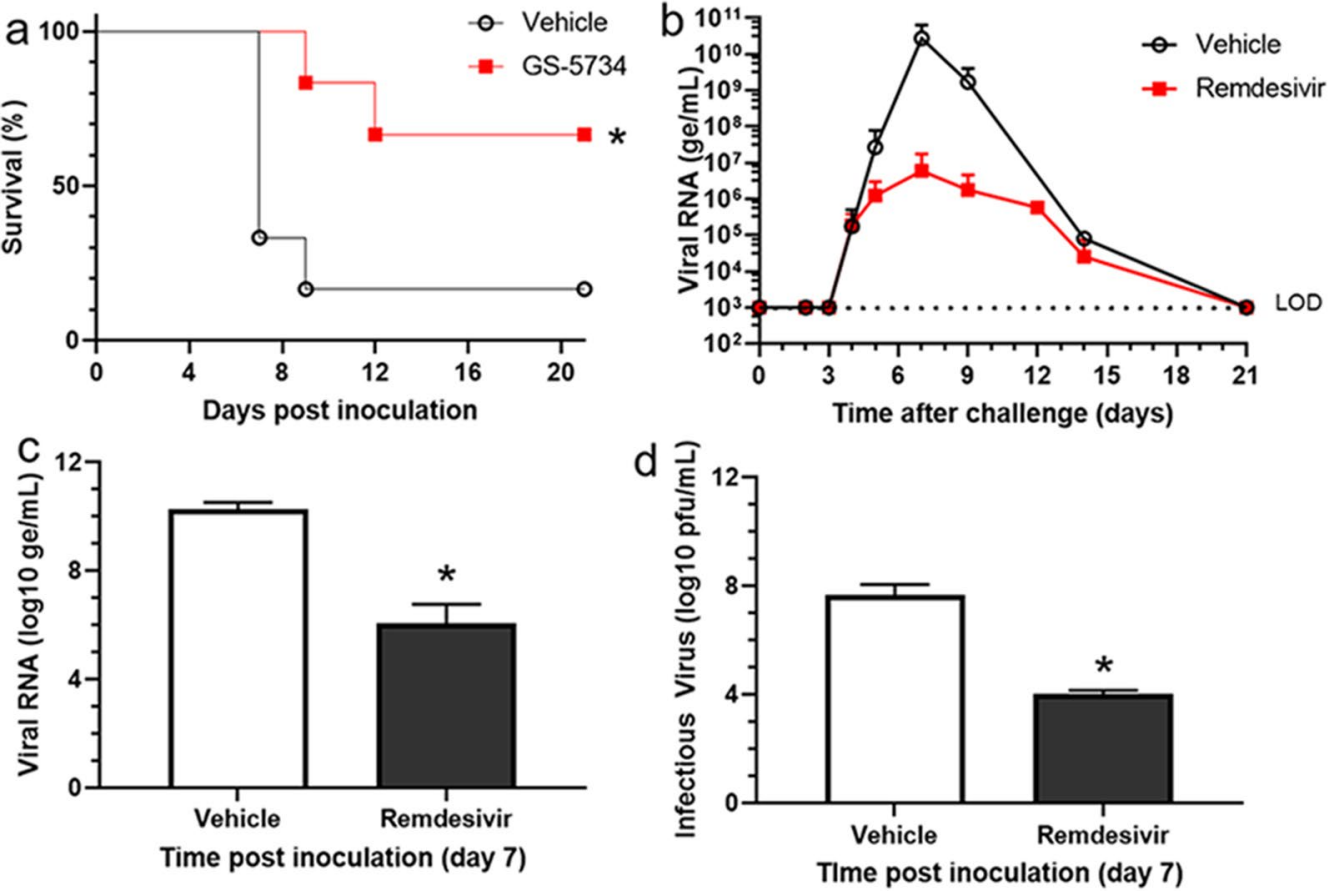
Survival and viremia. (**a**) Kaplan–Meyer survival curves show 1 of 6 survivors (17% survival) in the vehicle control group and 4 of 6 (67%) survivors in the remdesivir-treated group following aerosol delivery of EBOV, p = 0.032 using the log-rank Mantel-Cox test. (**b**) Group mean of plasma viral RNA concentrations indicated as genome equivalents/mL (ge/mL). Vehicle-treated NHPs are indicated by black open circles, and remdesivir-treated NHPs are indicated by red filled squares. *LOD* limit of detection. (**c**) Day 7 viral RNA levels. EBOV RNA (log_10_ ge/mL) is significantly lower in the remdesivir treatment (filled bar) compared to vehicle controls (open bar) on day 7, p = 0.029 (Wilcoxon rank-sum test). (**d**) Infectious EBOV levels. Infectious EBOV (log_10_ pfu/mL) is significantly lower with remdesivir treatment (filled bar) compared to vehicle controls (open bar) on day 7, p = 0.036 (Wilcoxon rank-sum test). Values represent the medians and interquartile ranges with statistically significant differences noted (*p < 0.05).

Five of six vehicle-treated animals were euthanized according to protocol-specified clinical criteria on day 7 PI (four animals) or day 9 PI (one animal; Fig. 1a). Two of six remdesivir-treated animals were euthanized; one remdesivir-treated nonsurvivor met the predefined euthanasia criteria (responsiveness score of 3 and altered serum chemistry) on day 9 PI, and the other (responsiveness score of 4 and altered serum chemistry) on day 12 PI. One vehicle-treated animal (17%) and four remdesivir-treated animals (67%) survived until the end of the study on day 42 PI. Animals in the remdesivir-treated group showed a significantly improved survival rate compared to the vehicle-treated control animals (p = 0.032; log-rank Mantel Cox).

### Viremia

Plasma viral RNA was first detectable on day 4 PI for three animals in the vehicle-treated group and four animals in the remdesivir-treated group (Supplementary Table S1). Beginning on day 5 PI, reduced viral RNA levels (genomes/mL; ge/mL) were observed in the remdesivir-treated group (~ 10^6^ ge/mL) compared to vehicle control group (~ 10^7^ ge/mL; Fig. 1b). By day 7 PI, viral RNA was detectable in the plasma of all 12 animals. Significantly lower plasma viral RNA levels were observed in the remdesivir-treated animals (1.35 × 10^6^ ge/mL) compared with the vehicle-treated animals (1.84 × 10^10^ ge/mL; p = 0.029 on day 7 PI; Fig. 1c), which correlated with the observed improvements in survival. The trend of reduced viral RNA levels in the remdesivir-treated group was observed on day 9 PI, but this reduction could not be tested for significance at later time points as there were too few survivors in the vehicle group for statistical evaluation. Viral RNA was undetectable in plasma from all surviving animals (including the surviving control animal) by day 21 PI (Supplementary Table S1).

Median serum infectious viral load on day 7 PI among untreated animals was 7.65 log_10_ plaque-forming units (pfu)/mL (Table 2). On day 7 PI, four of six remdesivir-treated animals had detectable serum infectious viral load; however, the value was quantifiable for only one animal (one of the two nonsurvivors in this group). The mean serum viremia in the remdesivir-treated animals (4.00 log_10_ pfu/mL) was more than 3 log_10_ lower than that of the vehicle control group (p = 0.036; Fig. 1d; Table 2). By day 21 PI, infectious viral load was undetectable in the serum of any of the surviving animals.

**Table 2:** Serum viremia (log_10_ pfu/mL).

Days post-inoculation	Parameter	Vehicle	Remdesivir	p-value
0	Median	< LOD	< LOD	N/A
	Interquartile range	< LOD	< LOD	
	n	6	6	
7	Median	7.65	4.00	0.036
	Interquartile range	< LOD–8.83	< LOD–4.62	
	n	6	6	
21	Median	< LOD	< LOD	N/A
	Interquartile range	< LOD	< LOD	
	n	1	4	

*LLOQ* lower limit of quantitation (4.00 log_10_ pfu/mL), *LOD* limit of detection (0 pfu/mL), *N/A* not applicable.

### Clinical signs

A modest elevation in body temperature up to 40 °C was observed from day 3 through day 9 PI. No significant differences were observed between the two treatment groups for body temperature or body weight. All animals remained free of clinical signs until day 6 PI (Supplementary Fig. S1). On day 6 PI, all five nonsurviving vehicle-treated animals began to display behavioral depression and deteriorating physical responsiveness, consistent with developing acute disease; these signs continued through the day of euthanasia. In the one surviving untreated animal, the onset of clinical signs consistent with EVD occurred on day 8 PI, with responsiveness returning to normal (score of 0) by day 15 PI. Among remdesivir-treated animals, the onset of disease signs occurred between days 8 and 10 PI. Among the four surviving remdesivir-treated animals, three showed relatively mild signs of disease, as indicated by low responsiveness scores (no greater than 2) until no later than day 13 PI. The two nonsurviving remdesivir-treated animals exhibited moderate to severe clinical disease signs (responsiveness scores of 3 or 4) on the day of euthanasia.

Pulse oximetry (%Sp02) evaluation of blood oxygen levels remained in the normal range (94–100%) for all vehicle-treated animals for each day of the study (Supplementary Fig. S2). This included five of six animals that succumbed to EVD. Similarly, all remdesivir-treated animals also displayed normal blood oxygen levels, with the exception of one nonsurvivor. This particular animal transitioned from a normal respiratory state on day 11 PI to a hypoxic state on day 12 PI prior to succumbing on day 13 PI.

Visual respiratory assessments appeared to be a more sensitive method for detection of EVD-mediated impact on the respiratory system. Only two of the six vehicle-treated animals displayed mild labored breathing (Supplementary Fig. S3). One was a nonsurvivor that scored 1 on day 7 PI and succumbed the next day, whereas the other was a survivor that scored a 1 on day 12 PI and subsequently returned to normal the following day. In contrast, five of six remdesivir-treated animals demonstrated mild labored breathing, which occurred between days 8 and 12 PI. Four of the five NHPs exhibited mild labored breathing for 1–2 days, which subsequently returned to normal (baseline) for the remainder of the study. The other animal scored a 1 on day 7 PI, progressed to severely labored breathing on day 12 PI, and succumbed the following day. The additional remdesivir-treated nonsurviving NHP did not show any respiratory signs prior to succumbing to EVD on day 10 PI.

### Clinical pathology

**Figure 2:**
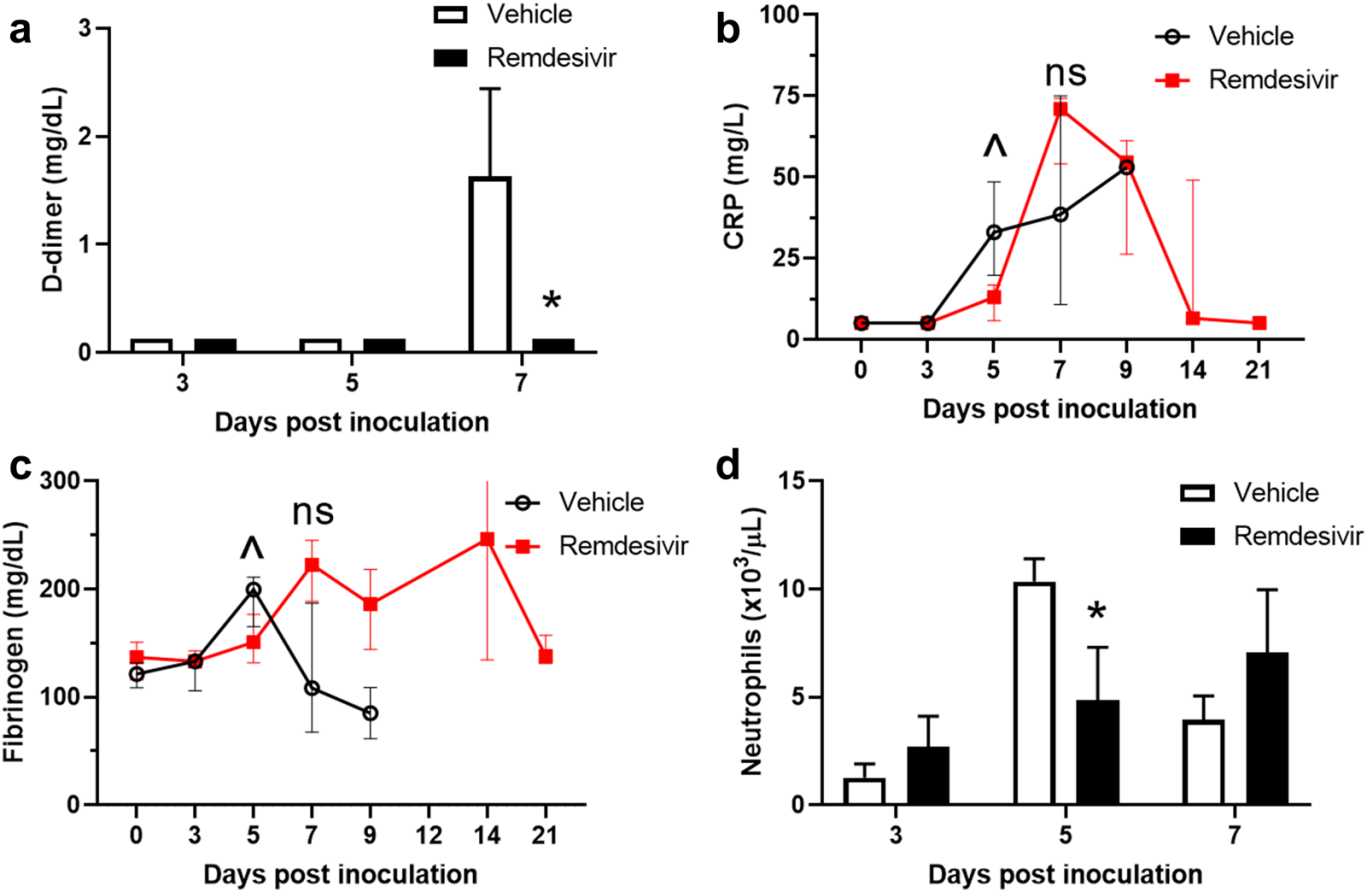
Systemic inflammatory responses. (**a**) D-dimer on day 7 PI is significantly reduced in remdesivir treatment group (filled bar) versus vehicle control group (open bar); p = 0.03 (Wilcoxon rank-sum test). Concentrations of (**b**) C-reactive protein (CRP) and (**c**) fibrinogen demonstrate trends (^p < 0.1) toward significant differences between remdesivir (red filled squares) and vehicle (black open circles) groups at day 5 PI. (**d**) Neutrophil counts are significantly different by day 5 PI, p = 0.05 (Wilcoxon rank-sum test). Values represent the group medians and interquartile ranges with statistically significant differences noted (*p < 0.05); *ns* not significant.

The clinical pathology observed was consistent with that previously documented in parenteral and aerosol EBOV exposure models of NHP infection. Early biomarker changes from baseline values indicated a systemic inflammatory response, coagulopathy, and hepatocellular damage. Some alterations were observed in the vehicle control cohort as early as day 5 PI and clearly by day 7 PI, which correlated with peak viremia. Remdesivir treatment, initiated at 4 days after EBOV exposure, ameliorated EVD-related biomarker activity. Systemic inflammation noted by D-dimer concentration was significantly improved by day 7 PI (Fig. 2a), whereas early elevations of other inflammatory markers, including C-reactive protein (CRP) and fibrinogen, trended toward reduction on day 5 PI (Fig. 2b,c). Neutrophil counts were also significantly reduced by remdesivir treatment at day 5 PI (Fig. 2d).

**Figure 3:**
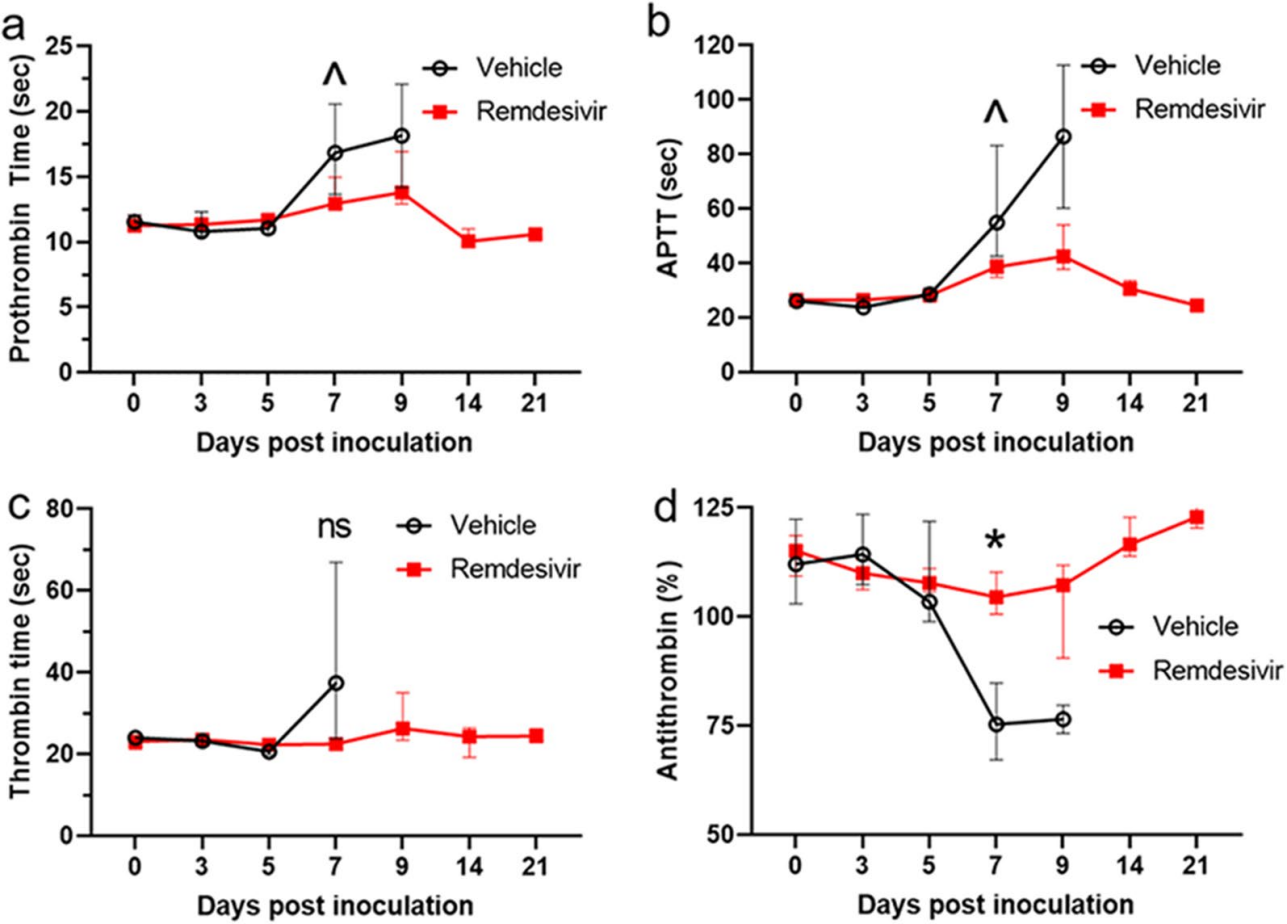
Clinical pathology. Trends toward increased clotting time were observed in vehicle treatment (black open circles) versus remdesivir treatment (red filled squares) groups for (**a**) prothrombin time, (**b**) activated partial thromboplastin time (APTT), and (**c**) thrombin time. (**d**) Anthithrombin (%) on day 7 PI is significantly reduced in control group (black open circles) versus remdesivir treatment group (red filled squares) with p = 0.03. Values represent the group medians and interquartile ranges, with *p < 0.05, ^p < 0.10; *ns* not significant.

Minimal peripheral coagulation abnormalities related to increased clotting times in plasma samples were observed in vehicle control rhesus monkeys, in accordance with an earlier report^21^. Compared to remdesivir-treated animals, vehicle-treated animals demonstrated trends toward abnormally prolonged clotting, as measured by increased prothrombin time and APTT at day 7 and/or day 9 PI (Fig. 3a,b). A non-statistically significant elevation of thrombin time in vehicle-treated monkeys was also observed at day 7 (Fig. 3c). A corresponding significant decrease in antithrombin % was observed at day 7 PI in this group. Remdesivir treatment appeared to stabilize this analyte in a statistically significant manner at day 7 PI (Fig. 3d).

**Figure 4:**
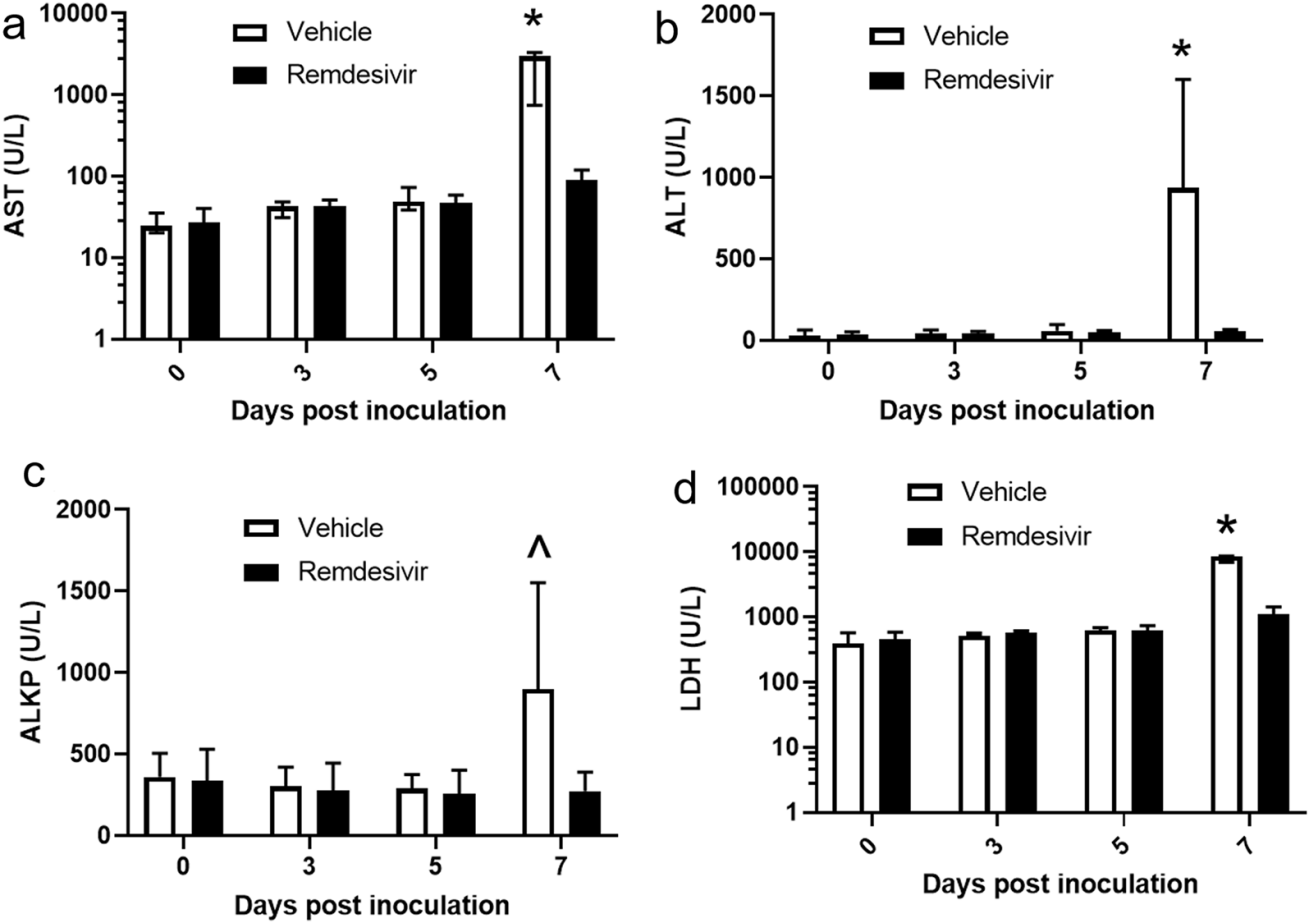
Clinical pathology alterations relating to hepatocellular and other systemic damage. A significant difference between the vehicle (open bar) and remdesivir (black bar) treatment groups was observed at day 7 PI for (**a**) AST (p = 0.05, Wilcoxon rank sum test), (**b**) ALT (p = 0.05, Wilcoxon rank-sum test), (**c**) ALKP trend (p = 0.09; Wilcoxon rank sum test), and (**d**) LDH (p = 0.04; Wilcoxon rank-sum test). Values represent the group medians and interquartile ranges, with *p < 0.05 and ^p < 0.1. *ALKP* alkaline phosphatase, *ALT* alanine aminotransferase, *AST* aspartate aminotransferase, *LDH* lactate dehydrogenase.

In vehicle-treated monkeys, increases in aspartate aminotransferase (AST), alanine aminotransferase (ALT), and alkaline phosphatase (ALKP) at day 7 PI were attributed to hepatocellular damage secondary to EBOV infection. Remdesivir treatment reduced elevations in AST and ALT in a statistically significant manner (Fig. 4a,b) with a trend toward reduction for ALKP (Fig. 4c). Lactate dehydrogenase (LDH) is abundant in the cytoplasm of many cells and is a relatively nonspecific marker of cellular damage. Interestingly, LDH was elevated in an early report of the EBOV AE model and has more recently been correlated with viral load and survival in NHP models of EBOV infection^25,36^. LDH concentration was induced nearly tenfold in vehicle-treated monkeys at day 7 PI and was significantly reduced by remdesivir treatment (Fig. 4d).

Additional clinical chemistry trends indicating fluid loss and compromised kidney function were also observed. Vehicle-treated animals demonstrated elevations of blood urea nitrogen (BUN), creatinine, and creatinine kinase at day 7 and day 9 PI (Supplementary Fig. 4a–4c). Remdesivir treatment trended toward reducing these increases, which subsequently normalized by day 14 PI. Characteristic trends comparing vehicle with remdesivir treatment for selected coagulation, serum chemistry, and hematology endpoints are listed in Supplementary Table S2.

### Gross findings

A number of gross lesions were found primarily with increased incidence and severity in the vehicle treatment group. These lesions were considered to be the result of exposure to EBOV and are consistent with the changes expected with EVD in rhesus monkeys. The vehicle control group presented with petechial, ecchymotic, or macular rash on face, axillary skin, chest, arms, abdomen, and legs. Blood was observed in the lumen as well as reddened and thickened mucosa in the gastrointestinal tract. The kidneys exhibited swollen and pale (yellow-tan) cortices while the liver was discolored (pale tan), swollen, and friable. The lungs were diffusely congested (dark red) and the tracheobronchial lymph nodes discolored (reddened) and enlarged.

The gross lesions in the remdesivir-treated group were limited to the liver, lung, and lymph nodes with occasional skin rash. Liver changes were restricted to nonsurvivors, and changes in the lung were mostly found in nonsurvivors (one surviving male had congested lungs). Enlarged lymph nodes were present in both survivors and nonsurvivors and correlated with a microscopic observation of hypertrophy of the germinal centers. Skin rash was present in nonsurvivors only.

### Microscopic findings

**Figure 5:**
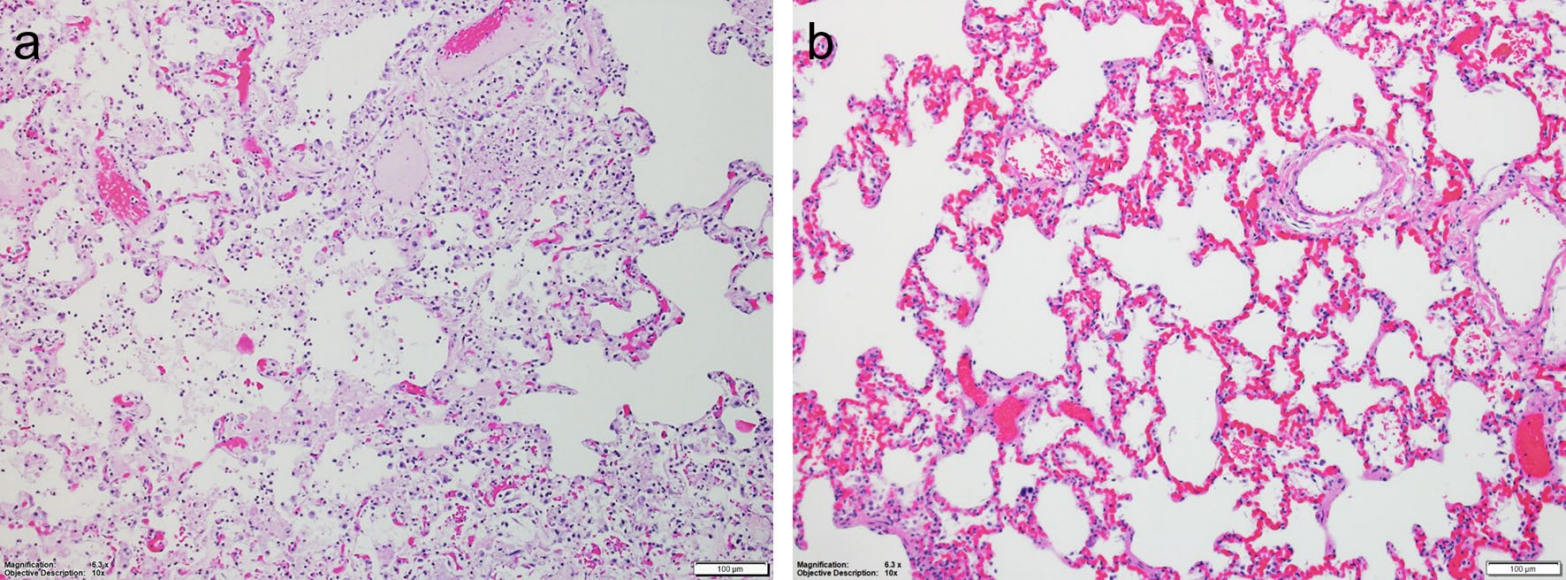
Lung histology. (**a**) Lung from vehicle-treated NHP (100× magnification). Focally extensive area of alveolar necrosis and edema are shown. (**b**) Lung from remdesivir-treated NHP survivor (100× magnification). Mild congestion is observed.

The vehicle control animals had decreased vacuolation of the adrenal gland cortex; tubular degeneration and/or necrosis of the kidneys; lymphoid depletion, hemorrhage in the marginal zone, fibrin deposition in the red pulp, and necrosis of the germinal center of the spleen. Hepatocellular degeneration or necrosis, as well as lung congestion and, in some animals, edema of the alveolus, alveolar necrosis, or thrombi in the lungs were also observed (Fig. 5a) Brain changes, including edema or hemorrhage of the choroid plexus and mixed cell inflammation in anterior and posterior chambers of the eye (in the untreated survivor only; Supplementary Fig. S5) were also noted. Importantly and in accordance with prior reports; lymphoid depletion, necrosis of germinal centers, or thrombus in the tracheobronchial lymph node were observed. In the remdesivir-treated NHPs, most of the EVD-associated changes were found in the two nonsurviving animals. These changes included, but were not limited to: tubular degeneration of the kidneys, lymphoid depletion and necrosis of germinal centers of the spleen, liver congestion and/or degeneration or necrosis of hepatocytes, and brain changes that included hemorrhage and mononuclear infiltration of the choroid plexus. A comparison of lung histological appearance is provided in Fig. 5b. Lung tissue from a vehicle-treated NHP (euthanized on day 9 PI) shows focally extensive areas of alveolar necrosis and edema, whereas lung tissue from a remdesivir-treated survivor shows mild congestion.

**Figure 6:**
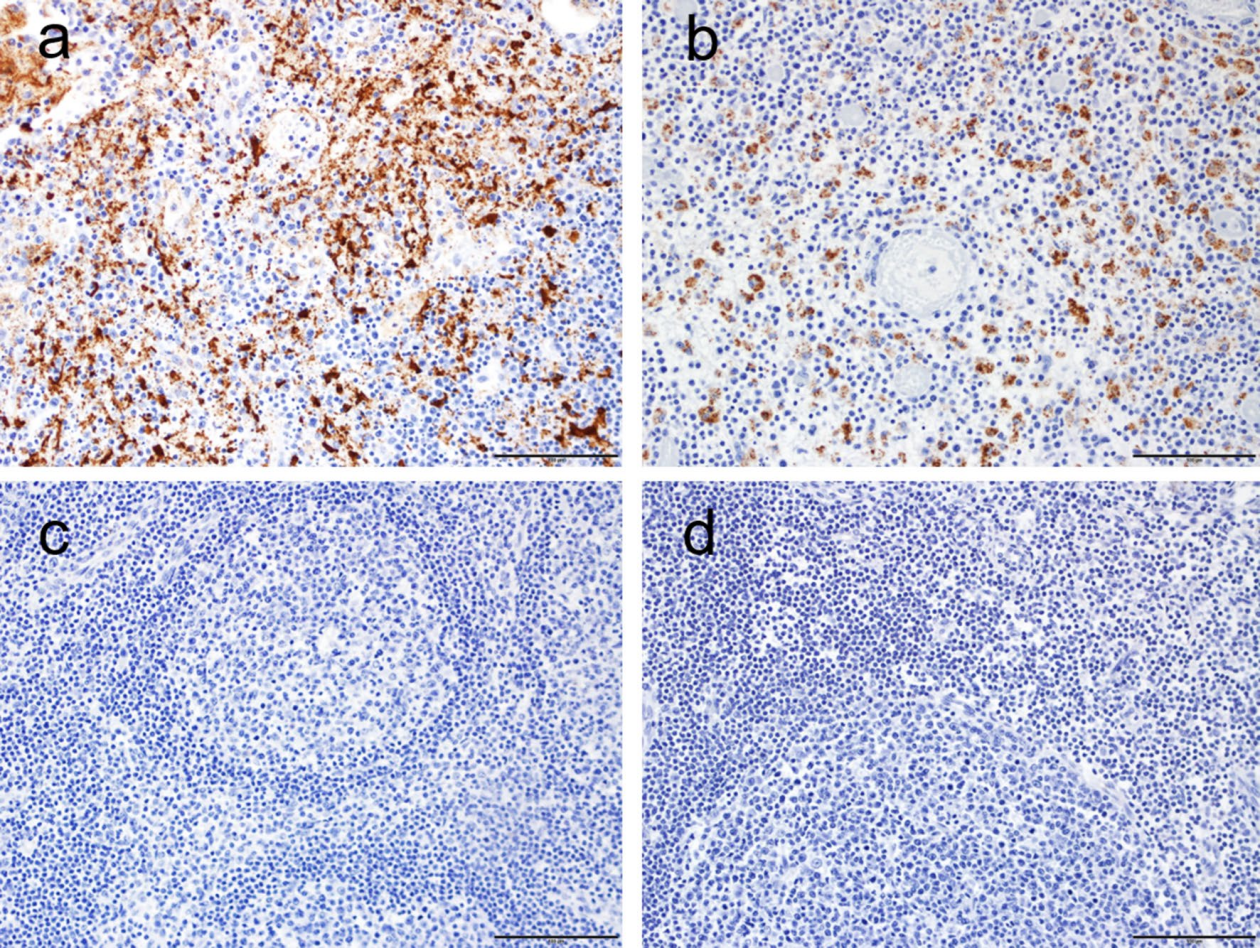
IHC staining of tracheobronchial lymph nodes**. **(**a**) Lymph node from an untreated nonsurviving NHP (100× magnification). Positive staining (brown) for EBOV viral antigen is shown. (**b**) Lymph node from a remdesivir-treated nonsurvivor (100× magnification). (**c**) Lymph node from an untreated survivor (100× magnification). No positive staining for EBOV antigens is shown. (**d**) Lymph node from a treated survivor (100× magnification). No positive staining for EBOV antigen is shown.

Immunohistochemistry (IHC) for viral antigen was positive in multiple organs in the nonsurviving animals of both groups. A nonsurviving female in the vehicle control group was positive for viral antigen only in the lung and tracheobronchial lymph node (Fig. 6a), both organs that had changes consistent with EBOV disease. A remdesivir non-survivor also demonstrate EOBV antigen in the tracheobronchial lymph node, although in a qualitatively less robust manner (Fig. 6b). Hypertrophy of the germinal centers in the tracheobronchial lymph nodes was observed in survivors 42 days PI, indicative of the immune response to infection (Fig. 6c,d). In the vehicle group survivor, viral antigen was detected only in the eye; this was associated with lesions in the eye of this animal (Supplementary Fig. 5). In the remdesivir-treated group, none of the surviving animals were positive for viral antigen. This pattern suggests that EBOV was largely cleared by day 12 PI.

## Discussion

To date, much of the EVD therapeutic landscape can be derived from the *pamoja tulinde maisha* (Swahili for “together save lives;” PALM) study: a randomized, controlled trial of the four investigational agents mAb114^37,38^, REGN-EB3^39,40^, remdesivir^34^, and ZMapp (another antibody cocktail of three monoclonal antibodies)^41^ for the treatment of patients with EVD^42^. In the PALM study control arm, the 28-day case fatality rate (CFR) in patients treated with ZMapp was 50% (84/169), which was statistically similar to the 53% (93/175) CFR in the arm treated with remdesivir. However, the 28-day CFR was significantly reduced in patients receiving either mAb114 (34%; 52/155) or REGN-EB3 (35%; 61/174). Despite these promising results for antibody-based therapeutics, substantial gaps remain in improving the outcomes of acute EVD, particularly for individuals with severe disease, and for prevention and treatment of viral persistence in immune-privileged sites. The latter may limit the overall utility of both vaccine and antibody-based therapeutic strategies^43,44^.

Novel, US Food and Drug Administration (FDA)-approved, treatment/prophylactic options for EVD include the rVSVΔG-ZEBOV-GP (V920) vaccine^45^, the mAb cocktail, REGN-EB3 (Inmazeb)^46^, and the mAb Ebanga (Ansuvimab-zykl)^8^. However, none of the treatments/prophylaxes currently approved by the FDA, have been evaluated in an aerosol NHP model of EBOV infection. In contrast, ZMapp^41^ was evaluated in an NHP aerosol model of EBOV infection. Initial studies demonstrated that ZMapp protects NHPs from death even when administered 5 days post IM exposure to EBOV. However, clinical evaluation of ZMapp during the 2013–2016 EVD epidemic failed to meet the pre-specified statistical threshold for efficacy^47^. In an aerosol model of EBOV infection in NHPs, treatment with ZMapp following virus exposure resulted in 100% survival in contrast to 0% survival observed in the control-treated animals. One ZMapp-treated NHP reached the lower limit of quantitation (LLOQ) of viral RNA (ge/mL) on day 28 PI in the two-infusion ZMapp treatment group, but no infectious virus (pfu/mL) was detected in either group. Lung effusion was observed in one NHP on day 14 PI and began to clear by day 28 PI. Histological examination revealed severe plural fibrosis and dense vascularized fibrous tissue in the lungs of two NHP survivors receiving two doses of ZMapp. These lung findings were not observed in NHPs in the ZMapp treatment studies involving EBOV exposure by the IM route. Such findings were also not observed in the remdesivir treatment group in the study described herein. The only observed changes in the lungs of remdesivir-treated animals were mild congestion and hypertrophy of the germinal centers in the tracheobronchial lymph nodes. These findings are indicative of the immune response to EBOV infection as it is known that EBOV dysregulates immune responses by both immune suppression and activation, although the role of these responses during infection is not completely understood^43^.

A recent report on the natural history of infection in rhesus monkeys exposed by the IM route to 431 pfu of EBOV/H.sapiens-tc/COD/1995/Kikwit provides a benchmark for comparison between aerosol and IM exposure routes in this model^22^. A distinguishing feature of AE, which is not found after IM exposure, is primary virus infection of the lymphoid tissues of the upper and lower respiratory tract^20^. In one previous study, inhalation of as low as 400 pfu of EBOV resulted in rapidly fatal disease 4–5 days post-exposure. Mild to moderate, patchy interstitial pneumonia with a bronchocentric pattern was observed on necropsy. EBOV antigens were detected in the airway epithelium, alveolar pneumocytes, pulmonary macrophages and pulmonary lymph nodes. Large amounts of extracellular viral antigen were also present in secretions on mucosal surfaces of the airways, oropharynx, and nose^25^. A study involving probable aerosol transmission of EBOV from piglets to crab-eating macaques demonstrated that infected NHPs developed interstitial pneumonia and had focal areas of alveolar hemorrhage and edema^48^. Although whether the pathologic changes were a result of primary respiratory diseases or secondary spread via hematogenous mode remains unknown, viral antigen staining in these macaques was similar to that observed in rhesus monkeys. In our study, congestion and thrombi in the lungs, as well as alveolar edema and necrosis, were observed in all or some of the nonsurviving NHPs. These pathological changes were absent or only mildly present in survivors. In tracheobronchial lymph nodes of nonsurvivors, hypertrophy, lymphoid depletion, and necrosis of the germinal centers were detected, the severity of which was higher in the vehicle group. Germinal center hypertrophy, however, was also observed in survivors. EBOV antigens were observed in the tracheobronchial lymph nodes in nonsurvivors but were not detected in survivors that recovered from infection. Together, these observations in NHPs illustrate variation in virulence and disease manifestation kinetics between aerosol and other exposure routes.

Qualitative differences in EVD following AE versus other exposure routes drive the interest in the efficacy of remdesivir in the rhesus model of infection. EBOV infection initiated in the lung differs from that initiated intramuscularly in that a different collection of cells are infected, resulting in differences in cytokines and chemokines released and in the timing of immune responses and immune suppressive events. Following AE, EBOV infects alveolar macrophages, some bronchiolar cells, and pneumocytes^20^. Alveolar macrophages of the lung are dense, adhering to epithelial cells through integrins (αvβ6); when triggered by infection, alveolar macrophages activate TGF-β^49^. Once activated, TGF-β suppresses macrophage release of cytokines and phagocytosis^50^. The macrophages interact with epithelial cells and T-cells and are under the influence of the tissue-specific microenvironment. The infected cells then migrate to different regional lymph nodes prior to systemic distribution of progeny virions^51^. Immune responses also differ following AE to EBOV versus other exposure routes. Mucosal IgA is elaborated in the lung in contrast to IgG, which is prevalent in nonmucosal infections. In summary, EBOV exposure via the aerosol route is qualitatively different from the IM route; consequently, the response to therapeutics may differ. Studies examining the impact of AE to EBOV on local immune responses in the lungs, the unique cell populations involved, and the timing of cytokine and chemokine release are greatly needed. Finally, response to vaccines and monoclonal antibody therapies following AE to EBOV have not been published. Such studies will enable understanding of possible qualitative differences in outcomes depending on routes of exposure.

Remdesivir was approved by the US FDA for the treatment of COVID-19 in September 2020^52^. In vivo, remdesivir treatment is efficacious in animal models of diseases caused by other viruses, such as MARV^35^, severe acute respiratory syndrome coronavirus (SARS-CoV), Middle East respiratory syndrome (MERS)-CoV^32,53^, and Nipah virus^30^. We demonstrate here, for the first time, that IV administration of remdesivir to animals exposed to aerosolized EBOV provides a survival benefit, significantly reduces viremia and infectious virus titer, and improves clinical scores of infected monkeys. Significant improvement in markers of inflammation (D-dimer, neutrophil count), coagulopathy (antithrombin %), and liver damage (AST, ALT) and other markers (LDH) were associated with remdesivir treatment. However, some biomarker characteristics of EVD were not significantly ameliorated by remdesivir (platelet counts, C-reactive protein and BUN concentrations). Such broad-spectrum activity of remdesivir, combined with the therapeutic benefits observed in remdesivir-treated NHPs following exposure to EBOV by other routes, underscore its therapeutic potential.

## Materials and methods

### Animals

All animal experiments were approved by the Institutional Animal Care and Use Committee of the United States Army Medical Research Institute of Infectious Diseases (USAMRIID) and carried out by certified staff according to the institution’s guidelines for animal use. The study was reported in accordance with ARRIVE guidelines (https://arriveguidelines.org).

Animals used in this study were experimentally naïve. Chinese-origin male and female rhesus monkeys were obtained from World Wide Primates. Animals were maintained at the test facility animal housing colony prior to assignment to the study and were transferred for acclimation to animal biosafety level 4 (ABSL-4) laboratory conditions at least 3 days prior to inoculation.

A total of 12 rhesus monkeys (6 males and 6 females) were subjected to experimental procedures. Each animal was exposed to EBOV/Kikwit and was then treated once daily with either remdesivir or vehicle. Animals were identified by vendor-applied tattoo and cage label. For reporting, in-study documentation, and sample identification purposes, animals were identified using the pre-study animal ID, cage label identifier, or the last four digits of the tattoo number. Animals were tested to ensure that they had no prior exposure to EBOV, herpes simian B virus, simian immunodeficiency virus, simian T-lymphotropic virus, or simian retrovirus. Additionally, any animals under treatment for an existing disease condition or injury were excluded as were any animals with a history of gastrointestinal disorders within 30 days prior to study initiation.

Animals were randomly assigned to treatment groups, stratified by sex and balanced by body weight and age. The order in which surviving animals were euthanized for necropsy at the end of the scheduled in-life phase was randomized. Personnel who administered remdesivir or vehicle treatments, routinely evaluated animal health, or assessed animals for euthanasia were experimentally blinded to the group assignment of all animals in the study. The criteria used as the basis for euthanasia of moribund animals were defined prior to study initiation and included magnitude of responsiveness, reduced body temperature, and/or specified alterations of serum chemistry parameters^54^.

Animals were housed individually in stainless steel cages with squeeze capabilities for handling. Primary enclosures conform to guidelines specified in the US Department of Agriculture Animal Welfare Act (9 CFR, Parts 1, 2, and 3) and as described in the *Guide for the Care and Use of Laboratory Animals* (ILAR publication, 2011, National Academy Press). Harlan Teklad 2050 monkey chow was provided daily to animals. The manufacturer routinely analyzes for maximal allowable concentrations of contaminants (e.g., heavy metals, aflatoxin, organophosphates, chlorinated hydrocarbons, and polychlorinated biphenyls). Water was provided ad libitum via an automatic watering system or water bottles.

### Challenge agent

The challenge agent used for this study was Ebola virus H. sapiens-tc/COD/1995/Kikwit (order *Mononegavirales*, family *Filoviridae*, species *Zaire ebolavirus*). The identity of this stock has been confirmed by agent-specific reverse transcription real-time polymerase chain reaction (RT-PCR) assay(s), as well as by sequencing on the Illumina MiSeq (150 bp paired-end format). This stock was determined to be 92.80% 7U variant.

A certified titer of 1.31 × 10^6^ pfu/mL was determined by agarose plaque titration assay on Vero E6 cells (BEI). This titer was used as the nominal concentration for all dilutions required to make the exposure inoculum.

### Aerosol challenge

The challenge dose for each animal was calculated from the minute volume determined with a plexiglass whole body plethysmograph box using Buxco XA software. The total volume of aerosol breathed was determined by the exposure time required to deliver the estimated inhaled dose. Anesthetized animals were exposed to a target dose of 100 pfu in the USAMRIID Head-Only Automated Bioaerosol Exposure System (ABES-II). Animals were exposed one at a time, in sequence of alternating treatment groups, until all animals were exposed. The challenge was generated using a Collison nebulizer to produce a highly respirable aerosol (flow rate 7.5 ± 0.2 L/minute). The system generates a target aerosol of 1–3 µm mass median aerodynamic diameter determined by TSI Aerodynamic Particle Sizer. Samples of the pre-spray suspension and of the aerosol collected from the exposure chamber using an all-glass impinger during each challenge, as well as an aliquot of the stock tube used to make the pre-spray suspension, were titred via plaque assay to determine the inhaled pfu for each animal.

### Remdesivir and vehicle

Remdesivir was formulated at Gilead Sciences in a vehicle consisting of water with 12% sulfobutylether-β-cyclodextrin (SBE-β-CD), pH adjusted to 3.5 using hydrogen chloride. The control vehicle, a solution of water with 12% SBE-β-CD, was supplied by Gilead Sciences as a formulated, ready-to-administer product. Remdesivir and the control vehicle were stored refrigerated at 2–8 °C.

Remdesivir was administered to one group of six animals once daily by slow bolus IV injection 4 days after virus exposure. Animals in this group received an initial loading dose of 10 mg/kg remdesivir followed by a once daily maintenance dose of 5 mg/kg on days 5–15 PI. A control group was included as a comparator study arm; six animals in this group received matching vehicle once daily on days 4–15 PI.

### Clinical methods

This study was conducted under ABSL-4 containment. Animal health status prior to challenge was monitored at least once daily. After aerosol exposure, all animals were monitored closely for signs of clinical disease and were assigned a responsiveness score of 0–4 at each observation. The responsiveness score uses a 5-point scale as follows: 0 (alert); 1 (decreased activity); 2 (mildly unresponsive, occasional prostration); 3 (moderate unresponsiveness, weakness); 4 (moderate to severe unresponsiveness, requires prodding, moderate prostration).

### Clinical pathology

Rectal temperature was collected daily. Body weight measurements were recorded on day 0, prior to viral exposure, and on days after inoculation when an animal was anesthetized by intramuscular injection of a solution containing ketamine (100 mg/ml) and acepromazine (10 mg/mL) at 0.1 mL/kg body weight for blood collection. Blood samples for clinical pathology assessments were collected from anesthetized animals by peripheral venipuncture (femoral vein or cephalic vein). Samples were collected into serum separator tubes, EDTA tubes, and/or sodium citrate tubes. Hematology analysis was conducted using an Advia 120 Hematology Analyzer (Siemens) with multispecies software. Serum chemistry samples were analyzed via a VITROS 350 Chemistry System (Ortho Clinical Diagnostics). Coagulation analyses were performed using a Sysmex CA-1500 or equivalent coagulation analyzer.

### Plasma viral RNA

Quantitative RT-PCR was performed as described previously^34^. Briefly, plasma samples were inactivated with TRIzol LS. Carrier RNA and QuantiFast High Concentration Internal Control (Qiagen) was spiked into the sample prior to extraction according to manufacturer’s instructions. Viral RNA was eluted in AVE buffer. Each extracted RNA sample was tested with the QuantiFast Internal Control RT-PCR RNA Assay (Qiagen) to evaluate the yield of the spiked-in QuantiFast High Concentration Internal Control. RT-PCR was conducted using an ABI 7500 Fast Dx. For quantitative assessments, the average of the triplicate ge per reaction were determined and multiplied by 800 to obtain ge/mL of plasma. The limits of quantitation for this assay are 8.0 × 10^4^ (4.9031 log_10_) – 8.0 × 10^11^ (11.9031 log_10_) ge/mL of plasma. For presentation purposes, EBOV RNA values reported as less than the limit of detection (LOD) were imputed as 3 log_10_ ge/mL; values reported as greater than LOD to less than the LLOQ were imputed as 4.9 log_10_ ge/mL.

### Serum infectious virus

*Plaque assays* were conducted using Vero cell monolayers in six-well plates. Cells were incubated with 100–200 µL of diluted serum in duplicate for 1 h ± 10 min, after which cells were overlaid with EBME-containing agarose. Plates were incubated under standard cell culture conditions for 7–9 days to allow for plaque formation. Secondary agarose overlay containing neutral red was applied to each well for 24–48 h to aide in plaque visualization and enumeration. Determination of pfu per volume of serum was conducted for each sample using the plaque number obtained from the least dilute serum sample for which plaques could be counted. The LLOQ for the plaque assay is 4.0 log_10_ pfu/mL of serum.

### Histology and immunohistochemistry

Tissues and organs were first examined in situ, then were dissected from the carcass. Tissues collected for histopathology and immunohistochemistry (IHC) were immersion-fixed in 10% neutral buffered formalin for a minimum of 28 days before removal from BSL-4 containment. The tissue samples were trimmed, routinely processed, and embedded in paraffin. The paraffin-embedded tissues were cut into 5-µm thick sections for histology. After the paraffin-embedded tissues were cut and placed on glass slides, they were deparaffinized, and stained with hematoxylin and eosin. To detect EBOV antigen in formalin-fixed paraffin-embedded tissues, IHC was performed using a cocktail of anti-EBOV antibodies (USAMRIID #702/703) for the detection of VP40 and GP with the Dako EnVision + System-HRP DAB kit according to manufacturer’s instructions.

### Statistical analysis

Analyses were performed using SAS software Version 9.4 TS1M5. For calculation purposes, the censored day of death for all surviving animals was set to 21 days. The proportion surviving in each group was compared using Fisher’s exact test. The mean time to death following challenge for each group was calculated. Rate of survival was calculated using the Kaplan–Meier method and compared between groups by log-rank test.

Wilcoxon rank-sum tests were used to compare chemistry, hematology, and coagulation parameters between groups on study days 3, 5, and 7. Correction for multiple pairwise comparisons within each parameter was made by the adaptive Holm method. The Wilcoxon rank-sum test was also used to compare serum viremia values between groups on study day 7. Assay values below the lower LOD (LLOD) were set to a value equal to the LLOD divided by the square root of 2 (LLOD/√2) prior to analysis. Assay values above the upper LOD were set to a value equal to the upper LOD prior to analysis.

The maximum viral RNA PCR (log_10_ ge/mL) value after challenge was calculated. The following algorithm was used to determine the average viral RNA PCR value after challenge. Briefly, the analysis window was determined by (1) starting at the first viral RNA (log_10_ ge/mL) value that is on or after the challenge date and (2) ending at the earliest of (i) observation end date, (ii) the last date with available RNA recorded for the animal, or (iii) the earlier date of two consecutive values that are “ < LOD.” Viral RNA PCR values recorded as “ < LOD” were set to the log_10_ value of the LLOD (38.07 ct) prior to analysis. RNA values that were recorded as “ > LOD < LLOQ” were set to a value equal to 4.9031 log_10_ ge/mL prior to analysis. Time-weighted average viral RNA value and maximum RNA value were compared between groups using Wilcoxon rank-sum tests.

Missing data were handled as missing at random, and no corrections for missing data were included in the analysis. Descriptive statistics include number of observations, median, and interquartile range. All significance tests performed were two-tailed. Significance levels were set at α = 0.05.

Figures were generated using GraphPad Prism software version 8.4.3 (GraphPad Software, San Diego, California USA).

## Supplementary Information

Supplementary Information.

## Data Availability

The data that support the findings of this study are available from the corresponding author upon reasonable request.
