# Supplementary material for: Remdesivir is efficacious in rhesus monkeys exposed to aerosolized Ebola virus
Source: Sci Rep. 2021 Sep 30;11:19458. doi: 10.1038/s41598-021-98971-0 (PMC8484580; doi:10.1038/s41598-021-98971-0)
Supplement: Supplementary file 1 — Supplementary Information. [file 41598_2021_98971_MOESM1_ESM.pdf]

Supplementary Tables and Figures

Supplementary Table S1.

Animal ID	Group Number	Treatment Description	day 0	day 2	day 3	day 4	day 5	day 7	day 9	day 12	day 14	day 21	day 28	day 35	day 42
1	1	Vehicle	<LOD	<LOD	<LOD	<LOD	5.87	9.66	9.53	.	.	.	.	.	.
2	1	Vehicle	<LOD	<LOD	<LOD	<LOD	5.51	6.66	6.95	--	>LOD <LLOQ	<LOD	<LOD	<LOD	<LOD
3	1	Vehicle	<LOD	<LOD	<LOD	5.92	8.12	10.36	.	.	.	.	.	.	.
4	1	Vehicle	<LOD	<LOD	<LOD	<LOD	6.23	11.00	.	.	.	.	.	.	.
5	1	Vehicle	<LOD	<LOD	<LOD	>LOD <LLOQ	7.20	10.33	.	.	.	.	.	.	.
6	1	Vehicle	<LOD	<LOD	<LOD	5.15	7.02	10.20	.	.	.	.	.	.	.
7	2	Remdesivir 10/5 mg/kg	<LOD	<LOD	<LOD	<LOD	<LOD	5.31	>LOD <LLO	--	4.99	<LOD	<LOD	<LOD	<LOD
8	2	Remdesivir 10/5 mg/kg	<LOD	<LOD	<LOD	5.54	6.14	6.00	>LOD <LLO	--	<LOD	<LOD	<LOD	<LOD	<LOD
9	2	Remdesivir 10/5 mg/kg	<LOD	<LOD	<LOD	5.28	5.84	6.53	6.54	5.76	.	.	.	.	.
10	2	Remdesivir 10/5 mg/kg	<LOD	<LOD	<LOD	5.67	6.67	7.46	6.83	.	.	.	.	.	.
11	2	Remdesivir 10/5 mg/kg	<LOD	<LOD	<LOD	<LOD	>LOD <LLOQ	6.12	>LOD <LLO	--	<LOD	<LOD	<LOD	<LOD	<LOD
12	2	Remdesivir 10/5 mg/kg	<LOD	<LOD	<LOD	5.08	5.75	6.01	>LOD <LLO	--	<LOD	<LOD	<LOD	<LOD	<LOD

Supplementary Table S1. Plasma viral RNA ($\log_{10}$ ge/mL). *LOD* lower limit of detection (Ct=38.07), *LLOQ* lower limit of quantitation (4.9031 $\log_{10}$ ge/mL), -- denotes an unscheduled event, period (.) denotes not collected due to deceased NHP (gray shading).

Supplementary Table S2.

Parameter	Day PI	Median value		
		Vehicle	Remdesivir	Adjusted p-value*
AAPT (sec)	3	23.8	26.55	0.24
	5	28.65	28.2	0.81
	7	54.9	38.6	0.06
	9	86.5	42.5	-
	14	-	30.6	-
	21	-	24.55	-
AT (%)	3	114.35	110	0.40
	5	103.45	107.8	0.40
	7	75.35	104.55	0.03
	9	76.5	107.25	-
	14	-	116.65	-
	21	-	122.9	-
D-dimer (mg/L)	3	0.13	0.13	1.00
	5	0.13	0.13	0.84
	7	1.63	0.13	0.04
	9	0.79	0.13	-
	14	-	0.13	-
	21	-	0.13	-

PT (sec)	3	10.8	11.35	0.29
	5	11.05	11.7	0.09
	7	16.85	12.95	0.16
	9	18.15	13.8	-
	14	-	10.05	-
	21	-	10.6	-
TT (sec)	3	23.25	23.55	0.75
	5	20.6	22.3	0.05
	7	37.4	22.5	0.21
	9	-	26.3	-
	14	-	24.3	-
	21	-	24.4	-
Albumin (g/dL)	3	4.15	3.75	0.15
	5	3.85	3.6	0.17
	7	3.05	3.45	0.07
	9	2.85	2.75	-
	14	-	2.7	-
	21	-	3.15	-
ALKP (U/L)	3	283.5	246	0.97
	5	269	231.5	0.97
	7	619	258.5	0.09
	9	553	273.5	-
	14	-	290.5	-

	21	-	444.5	-
ALT (U/L)	3	31	34.5	1.00
	5	47.5	46.5	1.00
	7	1036.5	58.5	0.05
	9	437	82	-
	14	-	61.5	-
	21	-	38.5	-
AST (U/L)	3	42.5	43	1.00
	5	49	46.5	1.00
	7	3136.5	90.5	0.05
	9	1984.5	182	-
	14	-	107	-
	21	-	27.5	-
BUN (mg/dL)	3	14.5	14	1.00
	5	14	15.5	0.87
	7	51.5	19	0.18
	9	64.5	25	-
	14	-	19	-
	21	-	12	-
Calcium (mg/dL)	3	10.1	9.65	0.04
	5	8.95	8.75	0.44
	7	5.9	8.95	0.03
	9	7.2	8.05	-

	14	-	8.4	-
	21	-	8.9	--
CRP (mg/L)	3	5	5	1.00
	5	33	13	0.09
	7	38.5	71	0.79
	9	53	54.5	-
	14	-	6.5	-
	21	-	5	-
Creatinine (mg/dL)	3	0.7	0.7	1.00
	5	0.7	0.7	1.00
	7	5.45	1.05	0.23
	9	3.55	1.2	-
	14	-	0.6	-
	21	-	0.5	-
CK (U/L)	3	682	633	0.79
	5	569	673.5	0.79
	7	2074.5	941.5	0.76
	9	4797.5	1652.5	-
	14	-	603.5	-
	21	-	150.5	-
GGT (U/L)	3	74	76	1.00
	5	75.5	71	1.00
	7	332	72	0.28

	9	128.5	62.5	-
	14	-	54.5	-
	21	-	78	-
Glucose (mg/dL)	3	83.5	76.5	0.49
	5	80	73	0.20
	7	42	77	0.20
	9	70.5	70	-
	14	-	74	-
	21	-	64	-
LDH (U/L)	3	519	576.5	0.64
	5	631	617.5	0.70
	7	8600	1098.5	0.04
	9	-	2071	-
	14	-	1869	-
	21	-	736	-
Abs Lymphocytes ($x10^3$ cells/□L)	3	2.78	3.35	0.16
	5	1.59	2.34	0.20
	7	2.08	1.37	0.49
	9	1.82	1.975	-
	14	-	4.25	-
	21	-	5.63	-
Abs Neutrophils ($x10^3$ cells/□L)	3	2.46	2.71	0.49
	5	10.34	4.86	0.05

	7	3.93	7.07	0.16
	9	2.56	2.55	-
	14	-	3.84	-
	21	-	6.45	-
Platelets (x10^3 cells/□L)	3	283.5	314.5	1.00
	5	258	278.5	1.00
	7	131	143.5	1.00
	9	43.5	106	-
	14	-	296.5	-
	21	-	500.5	-
WBC (x10^3 cells/□L)	3	5.30	6.96	0.09
	5	12.39	8.66	0.07
	7	6.02	9.04	0.20
	9	4.62	5.23	-
	14	-	8.76	-
	21	-	12.07	-

Supplementary Table S2. Statistical summary of selected clinical pathology parameters. *AAPT* activated partial thromboplastin time, *Abs* absolute, *ALKP* alkaline phosphatase, *ALT* alanine aminotransferase, *AST* aspartate aminotransferase, *AT* antithrombin, *BUN* blood urea nitrogen, *CK* creatinine kinase, *CRP* C-reactive protein, *GGT* gamma glutamyl transferase, *LDH* lactate dehydrogenase, *PT* prothrombin time, *TT* thrombin time, *WBC* white blood cell. * Wilcoxon rank-sum test with adjustment for multiple comparisons for each remdesivir treatment vs vehicle

group on days 3, 5, and 7 (n= 6/group). Vehicle group days 0–7: n=6; Day 9: n=2, Day 14: n=1 (median value not shown). Remdesivir group Days 0-9, n= 6, and Days 14-21: n=4.

Supplementary Figures and Figure Legends

Supplementary Figure S1.

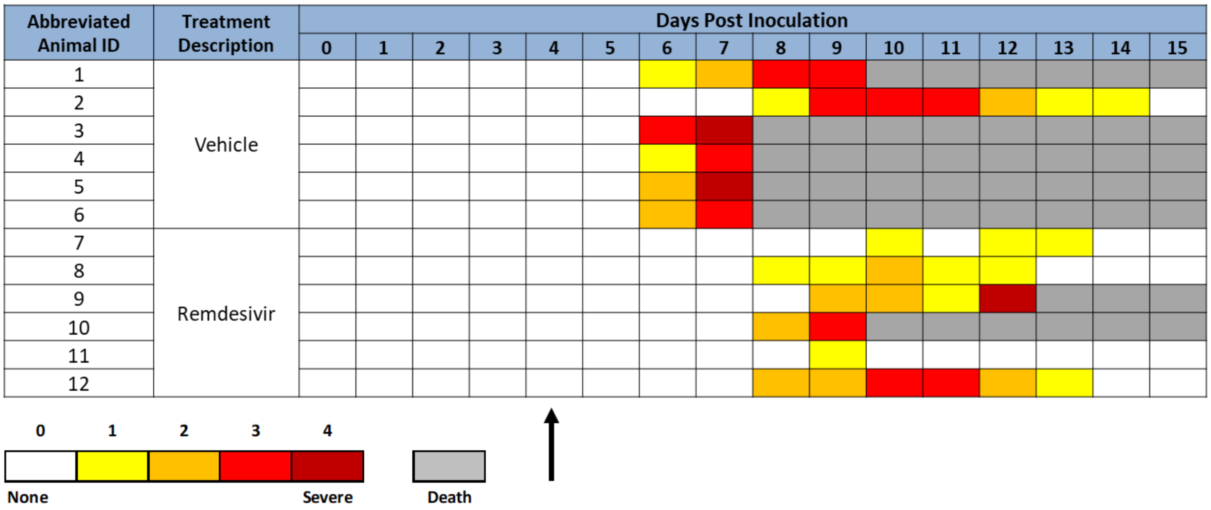

Supplementary Figure S1. Daily clinical assessments with a subjective clinical disease score denoted by increasing values (0–4) based on responsiveness, posture, and activity. Maximum daily scores were converted to a color code with darker colors indicative of more severe disease signs. The grey boxes indicate death. Arrow indicates initiation of remdesivir treatment. The schematic was truncated to emphasize clinical scores during the acute disease phase, with none of the surviving animals exhibiting clinical disease signs outside of the times that are shown.

Supplementary Figure S2.

Abbreviated Animal ID	Treatment Description	Days Post Inoculation														
		0	2	3	4	5	6	7	8	9	10	11	12	13	14	15
1	Vehicle	98	99	99	99	94	100	97	99	99						
2		95	100	99	99	99	99	98	99	100	99	97	96	96	96	98
3		98	99	97	99	98	99	99								
4		99	99	99	99	99	98	99								
5		99	98	99	100	99	99	95								
6		100	98	99	98	97	99	99								
7	Remdesivir	100	99	97	100	99	100	99	98	95	98	100	100	99	99	99
8		98	95	97	99	98	99	94	97	99	99	100	99	98	99	99
9		95	99	98	100	95	96	99	99	98	96	96	85			
10		99	99	99	99	99	99	99	99	97						
11		96	99	96	100	99	99	98	99	100	99	100	99	99	100	99
12		99	99	97	99	99	99	98	98	99	99	99	99	99	99	98

94-100% 85-93% <85%

Normal Hypoxic Severe Death

Supplementary Figure S2. Blood oxygen saturation measured via pulse oximetry. Blood oxygen saturation was evaluated with a rectal probe whenever an animal was anesthetized for blood collection or other assessments. Oxygen saturations (%Sp02) are shown as numerical values and the ranges for normal, hypoxic, and severe hypoxia conditions are indicated by white, orange and red shading. The grey boxes indicate death. Arrow indicates initiation of remdesivir treatment. The schematic was truncated to emphasize pulse oximetry values during the acute disease phase, with all animals exhibiting normal oxygen saturation through study end.

Supplementary Figure S3.

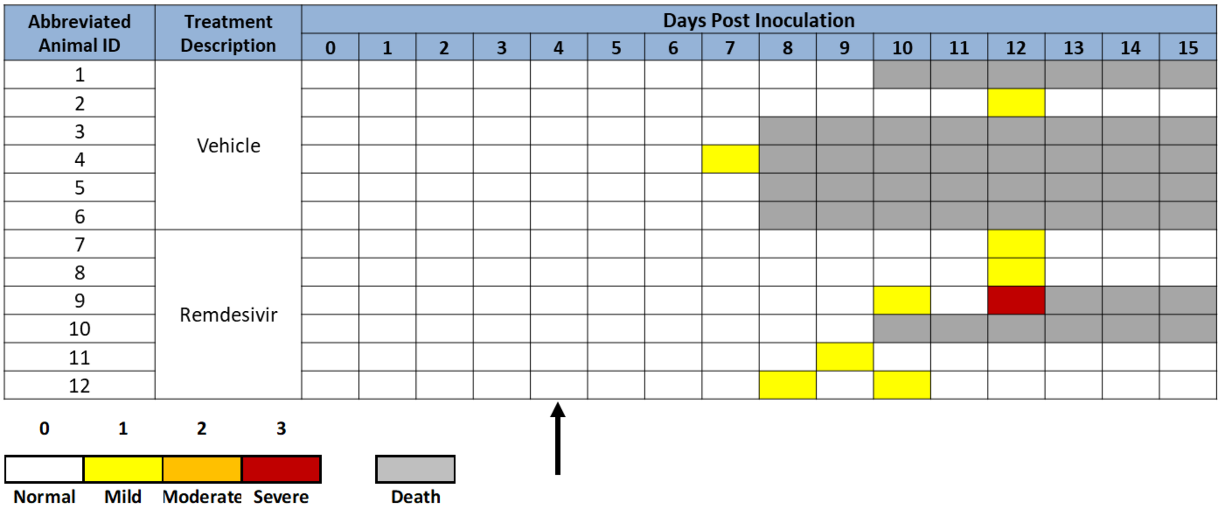

Supplementary Figure S3. Daily respiratory assessments with subjective respiratory score denoted by increasing values (0–4), which were converted to a color code with darker colors indicating more severe, labored breathing. The grey boxes indicate death. Arrow indicates initiation of remdesivir treatment. The schematic was truncated to emphasize respiratory scores during the acute disease phase, with none of the surviving animals exhibiting respiratory signs after day 13 PI.

Supplementary Figure S4.

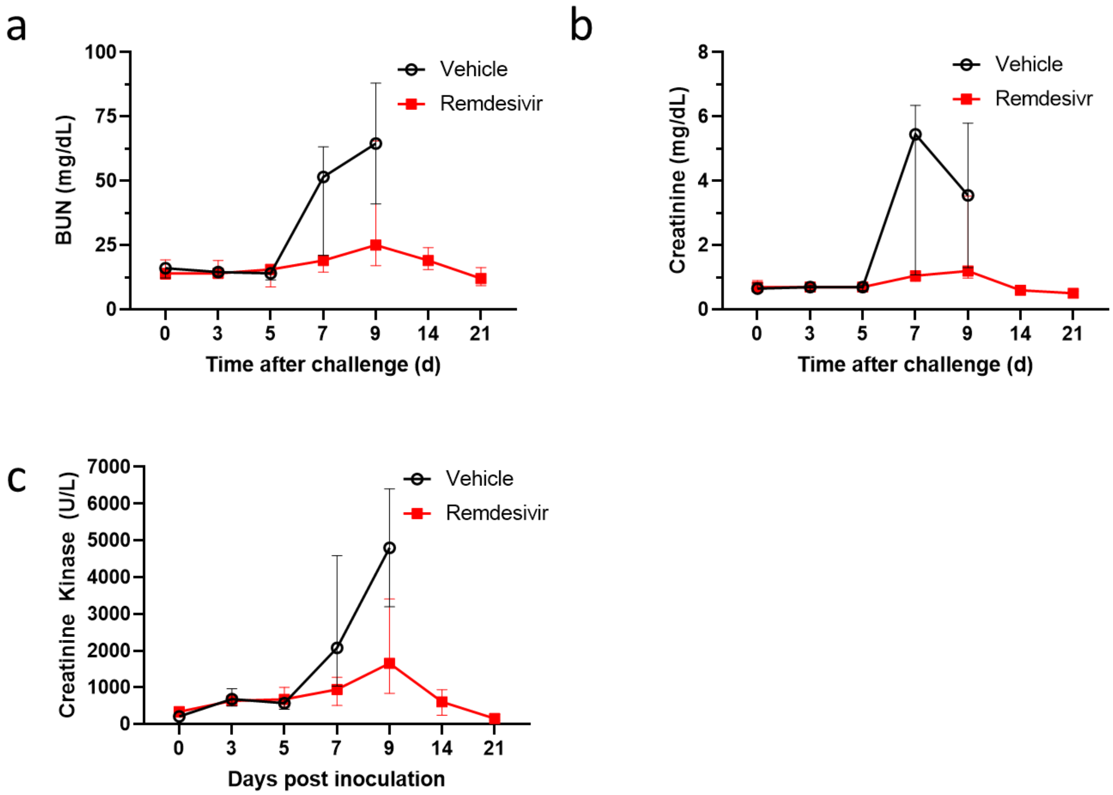

Supplementary Figure S4. Clinical pathology trends indicative of fluid loss. Vehicle treatment group (black open circles) yielded increasing analyte values between days 5 and 9 PI relative to remdesivir treatment group (red filled squares). A trend, but not a significant difference between the two groups, was observed for **(a)** blood urea nitrogen (BUN), **(b)** creatinine, and **(c)** creatinine kinase. Values represent the medians and interquartile ranges.

Supplementary Figure S5.

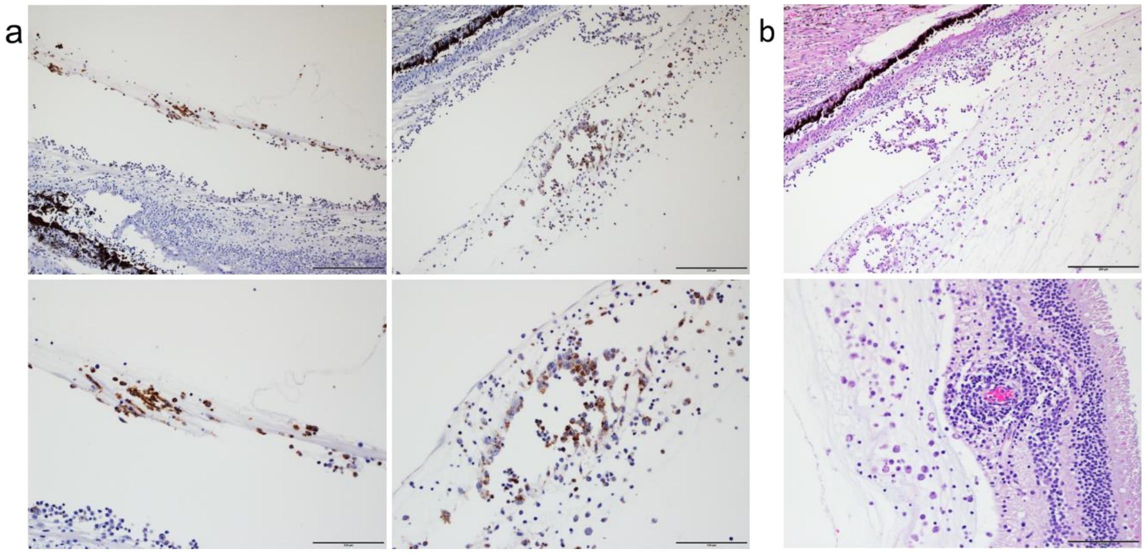

Supplementary Figure S5. IHC Images from the eye of survivor animal #2 in the vehicle group. **(a)** IHC images showing EBOV antigen staining (brown) in the eye (100X). Bottom panel shows high-magnification images (200X). Nuclei was stained blue by hematoxylin. **(b)** Histology (hematoxylin and eosin) staining showing inflammation in the eye. Top: uveitis (100X); bottom: vasculitis (200X).
